# Mechanisms of Regulated and Dysregulated CARD11 Signaling in Adaptive Immunity and Disease

**DOI:** 10.3389/fimmu.2018.02105

**Published:** 2018-09-19

**Authors:** Jacquelyn R. Bedsaul, Nicole M. Carter, Katelynn E. Deibel, Shelby M. Hutcherson, Tyler A. Jones, Zhaoquan Wang, Chao Yang, Yong-Kang Yang, Joel L. Pomerantz

**Affiliations:** Department of Biological Chemistry, Institute for Cell Engineering, The Johns Hopkins University School of Medicine, Baltimore, MD, United States

**Keywords:** CARD11, Bcl10, MALT1, primary immunodeficiency, lymphoma, T cell receptor, B cell receptor

## Abstract

CARD11 functions as a key signaling scaffold that controls antigen-induced lymphocyte activation during the adaptive immune response. Somatic mutations in CARD11 are frequently found in Non-Hodgkin lymphoma, and at least three classes of germline CARD11 mutations have been described as the basis for primary immunodeficiency. In this review, we summarize our current understanding of how CARD11 signals, how its activity is regulated, and how mutations bypass normal regulation to cause disease.

## Introduction

Our understanding of the immune system has benefited greatly from the study of the molecular circuitry in lymphocytes that governs antigen recognition and the cellular and systemic response to pathogens. This circuitry is elaborate, highly regulated, sensitive, and specific. As the study of immune cell signal transduction has highlighted networks of molecules that translate antigen sensing into lymphocyte action, it has also revealed how the dysregulation of signaling machinery can precipitate disease, including immunodeficiencies, autoimmunity syndromes, leukemia, and lymphoma.

**Figure 1:**
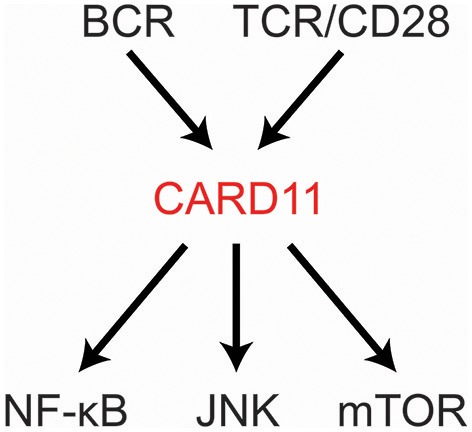
CARD11 relays signaling from antigen receptors to NF-κB, JNK, and mTOR.

CARD11 is a fascinating, multi-domain scaffold protein that plays a key role as a signaling hub during the adaptive immune response. Underscoring its importance, the CARD11 gene is extremely intolerant to loss-of-function (LOF) mutation or genetic variation in the human population (1, 2). Since its discovery in 2001 (3), many studies have revealed its obligate role in antigen-mediated lymphocyte activation and its susceptibility to mutations that can cause immunodeficiency or contribute to the development of lymphoma. CARD11 is best understood at present as a signal integrator that translates B cell receptor (BCR) and T cell receptor (TCR) triggering into the activation of NF-κB, JNK, and mTOR (Figure 1). Several excellent reviews have summarized the biological roles of CARD11 gleaned from mice and humans deficient in CARD11 or its signaling partner proteins (4–8). Here we will review our current understanding of (1) how the structure of CARD11 allows it to function as a signal-responsive scaffold; (2) how CARD11 orchestrates downstream signaling events; (3) the mechanisms that limit or terminate CARD11 signaling activity; (4) how gain-of-function (GOF) disease-associated CARD11 mutations bypass normal regulation; and (5) how LOF disease-associated CARD11 mutations disrupt CARD11 activity.

## Overview

**Figure 2:**
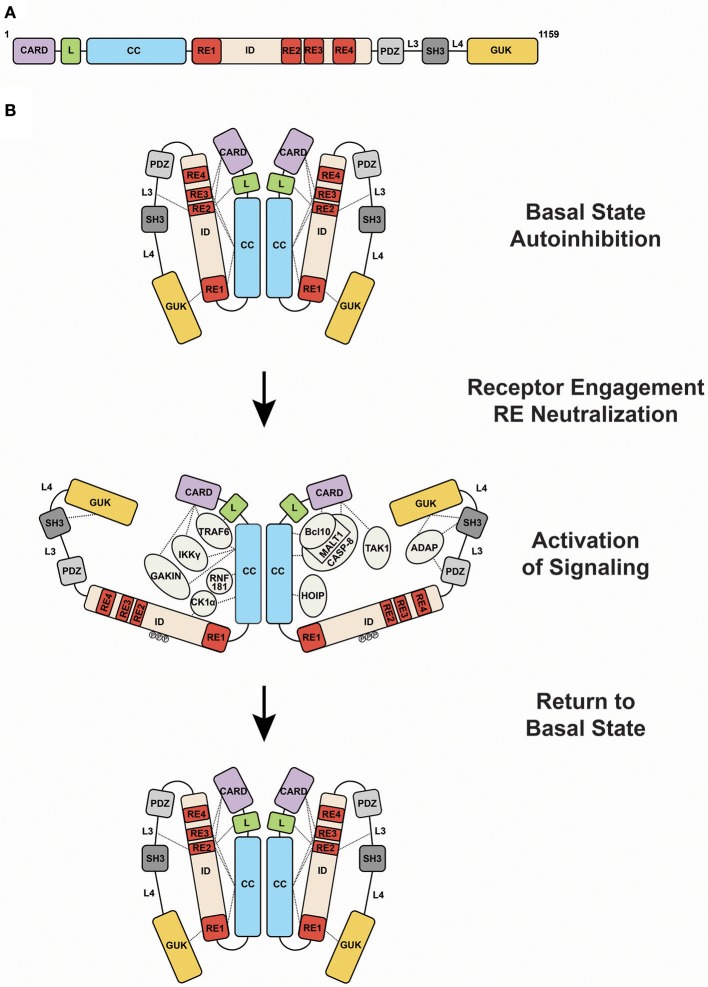
Activation of CARD11 activity during antigen receptor signaling. **(A)** Domain structure of CARD11. **(B)** Transition of CARD11 from closed, inactive to open, active states and back again. Dashed lines indicate mapped protein-protein interactions. Abbreviations: CARD, Caspase Recruitment Domain; L, Latch; CC, coiled-coil; ID, Inhibitory Domain; PDZ, PSD-95/Discs-Large/ZO-1 domain; SH3, Src Homology 3 domain; GUK, Guanylate Kinase domain; RE1, Repressive Element 1; RE2, Repressive Element 2; RE3, Repressive Element 3; RE4, Repressive Element 4; L3, Linker 3; L4, Linker 4.

CARD11 can be thought of as a string of protein-protein interaction domains, each of which presents surfaces for the binding and regulation of interaction targets (Figure 2A). The protein interacts with more than 20 different proteins during signaling, and it has evolved to present its interaction surfaces dynamically in response to signaling inputs (Figure 2B). Prior to receptor engagement the protein exists in a closed, inactive state. Receptor engagement leads to the conversion of CARD11 to an open, active scaffold that binds signaling partners. Cofactor binding leads to the generation of downstream signaling intermediates that activate the IKK complex, a central target in the canonical NF-κB signaling pathway, as well as JNK2, and mTOR. Following activation, binding partners dissociate from CARD11 and the protein returns to the inactive state. This cycle, from closed to open and back to closed states, is thought to occur over the course of approximately 60 minutes following BCR or TCR engagement.

## Maintenance of the closed inactive state

Like many signaling proteins that promote cellular proliferation and activation and whose signaling could be dangerous if dysregulated, CARD11 contains an internal autoinhibitory domain that keeps CARD11 inactive in the absence of receptor triggering (9–11). This domain, termed the Inhibitory Domain (ID), is located in the primary sequence between the Coiled-coil and PDZ domain (Figure 2A). Remarkably, the autoinhibitory action of the ID is accomplished by four small Repressive Elements (REs), ranging in size from 11 to 53 amino acids, that function cooperatively with redundancy (12, 13). Other than a short 5-residue region of homology between RE2 and RE3, the four REs do not resemble each other. While the mutation of any single RE has little to no effect on basal CARD11 signaling, their combinatorial mutation cooperatively increases activity, and the simultaneous mutation of all four leads to a remarkable 640-fold enhancement of activity (12). The four REs function together to prevent cofactor binding to CARD11 prior to receptor engagement, but it is not completely clear how they do so. RE1, RE2, and RE3 interact with other CARD11 domains, including the CARD, LATCH, Coiled-coil, L3, and GUK, with somewhat overlapping intramolecular specificities, presumably to maintain CARD11 in a conformation in which protein-interaction surfaces of the CARD and Coiled-coil are inaccessible (13). However, RE4, which may be the most potent inhibitory element, does not appear to participate in intramolecular interactions and likely functions in cooperation with the other REs through an unknown mechanism, possibly via the recruitment of a repressor *in trans*.

## Receptor-induced activation of CARD11 scaffold potential and conversion to the open active state

Antigen receptor engagement by antigen in the appropriate context leads to a cascade of signaling events upstream of CARD11 that have been extensively reviewed (14). At present, it appears that the inputs that CARD11 receives from upstream signaling consist of phosphorylation events within the ID (9, 10, 15–17). Serines 564, 567, 577, and 657 all appear to be phosphorylated as a result of antigen receptor signaling. Somehow the modification of these residues leads to sufficient neutralization of RE activity to allow CARD11 signaling cofactors to bind and signaling events to ensue. Serines 564 and 657 appear to be targeted by PKCβ in B cells and PKCθ in T cells, while serine 567 appears to be phosphorylated by IKKβ. The kinase that targets serine 577 has not yet been reported. Additional phosphorylation events may also be important for maximal CARD11 activity, depending on cellular context (18–20). The phosphorylation-mediated activation of CARD11 scaffold activity may occur in a step-wise manner, with an initial step that elicits some IKKβ kinase activity and a subsequent step in which IKKβ phosphorylation of serine 577 promotes full scaffold activity (15, 21). Precisely how the phosphorylation events convert CARD11 into an active scaffold remains mysterious. It is possible that phosphorylation induces a conformational change in CARD11 that disallows RE-mediated inhibitory intramolecular interactions. Alternatively, the phosphorylated residues may be recognized by an unknown factor *in trans* that actively prevents cooperative RE action.

## Receptor-induced recruitment of signaling cofactors

Upon activation, the protein interaction surfaces of CARD11 become accessible for binding to a variety of proteins (Figure 2B). Bcl10 was the first protein shown to bind CARD11, with MALT1 recruited indirectly through Bcl10. These observations led to the notion of a “CBM complex,” which is a misleading misnomer because it ignores the binding of other critical factors to CARD11. Bcl10 requires both the CARD and Coiled-coil domain of CARD11 for its recruitment at physiological levels of expression (11). The CARD and Coiled-coil are also required for the recruitment of TRAF6, IKKγ, and Caspase-8 (11). The CARD alone is required for TAK1 binding (11), while the Coiled-coil alone is required for binding to HOIP, the catalytic subunit of the Linear Ubiquitin Chain Assembly Complex (LUBAC) (22). CK1α is recruited through the Coiled-coil and ID (23). The preponderance of protein interactions occurs through the N-terminal half of CARD11 that includes the CARD, LATCH, and Coiled-coil, although the C-terminal PDZ-SH3-MAGUK region can bind the ADAP adapter (24) and AIP (25). In T cells, the site of CARD11 complex assembly appears to be the immunological synapse (26, 27).

In addition to the intramolecular interactions mediated by the REs in the ID, two other CARD11 regions have been shown to mediate CARD11-CARD11 interactions. The Coiled-coil domain, predicted to form four discontinuous regions with coiled-coil character, mediates assembly of CARD11 into an oligomer of undetermined stoichiometry. The SH3-GUK domain tandem has also been shown to participate in modular inter- and intramolecular binding interactions that appear to be required for higher order clustering of CARD11 visible via microscopy (28).

## CARD11-dependent signaling events in antigen receptor signaling

The transient recruitment of cofactors to CARD11 ultimately leads to the activation of the IKK complex in the canonical NF-κB activation pathway. As it assembles cofactors into complexes, CARD11 orchestrates ubiquitinylation and phosphorylation events that somehow work together to promote IKK kinase action on inhibitory IκB proteins that tether NF-κB in the cytoplasm. The phosphorylation of IκBs promotes their ubiquitinylation and degradation by the proteasome, which allows NF-κB to stably translocate to the nucleus to bind target genes.

## Bcl10 polyubiquitinylation

The signal-induced recruitment of Bcl10 and HOIP to CARD11 allows HOIP (enzyme) to conjugate Bcl10 (substrate) with linear ubiquitin chains, to produce LinUb_n_-Bcl10 (22). LinUb_n_-Bcl10 then binds the IKK complex through the UBAN domain of IKKγ (22) in an interaction thought to be required for IKK complex kinase activation (29). LinUb_n_-Bcl10 is a signaling intermediate that determines the extent of NF-κB activation downstream of CARD11 triggering. For a range of hyperactive CARD11 variants (see below), the levels of LinUb_n_-Bcl10 produced by each variant correlates with the degree of NF-κB activation it achieves (22). Signaling through LinUb_n_-Bcl10 accounts for 50-60% of the signaling output of CARD11 to NF-κB. In addition to CARD11, TCR-induced LinUb_n_-Bcl10 generation also requires MALT1, but not the SHARPIN subunit of LUBAC (22).

Bcl10 is also conjugated with K63-linked ubiquitin chains during antigen receptor signaling (30, 31) to form Ub_n_(K63)-Bcl10. This modification, mediated by cIAPs, has been shown to be a prerequisite for linear ubiquitinylation of Bcl10 in the context of chronic BCR signaling (32).

## IKKγ polyubiquitinylation

CARD11 facilitates the polyubiquitinylation of IKKγ with K63-linked chains in response to antigen receptor triggering (33), which is accomplished by a MALT1-associated E3 ligase activity (34). Linear ubiquitinylation of IKKγ has also been implicated in the antigen receptor pathway (35, 36). Since CARD11 recruits both HOIP and IKKγ upon activation, CARD11 likely facilitates LUBAC action on IKKγ.

## MALT1 polyubiquitinylation

MALT1 is also conjugated with K63-linked ubiquitin chains during signaling, which facilitates its interaction with IKKγ (37); however, MALT1 polyubiquitinylation has not been formally shown to require CARD11. TRAF6 has been implicated as the E3 ligase for this process, and its recruitment by CARD11 may promote its action on MALT1.

## MALT1 proteolytic action

CARD11 is also required for MALT1 protease activity on several targets, including Bcl10 and MALT1 itself (7, 38–40). MALT1 cleaves the inhibitory deubiquitinase A20 (41, 42) to limit its removal of polyubiquitinylated MALT1, thereby extending the time-course of NF-κB activation. MALT1 also cleaves the NF-κB subunit RelB, limiting its potential to repress transcriptional activation by RelA and c-Rel (43). In addition, MALT1 cleaves the HOIL-1L subunit of LUBAC and in so doing limits the degree of NF-κB activation downstream of CARD11 (36, 44, 45). MALT1 has also been shown to cleave the deubiquitinase CYLD to maximize NF-κB and JNK activation (46–48). CARD11 may promote MALT1 protease activity by activating enzymatic potential, by recruiting enzyme to substrate, or both, but further studies are required to define mechanisms.

## MTOR activation

The activation of mTOR downstream of TCR engagement also requires CARD11, in a role independent of IKK complex activation (49, 50). CARD11 signaling to mTORC1 depends upon the proteolytic activity of MALT1 (49) and the rapid uptake of glutamine through the ASCT2 glutamine transporter (50). CARD11 signaling promotes ASCT2 expression and ASCT2 associates with CARD11, Bcl10, and MALT1 during signaling, suggesting an active regulation of transporter activity (50).

## JNK activation

CARD11 is also required for the activation of JNK signaling following antigen receptor triggering (51, 52), in a manner that also requires Bcl10 and MALT1. For JNK activation, CARD11 appears to promote Bcl10 oligomerization, followed by the binding of Bcl10 to TAK1, MKK7, and JNK2, which is thought to engage this MAP kinase cascade for JNK2 activation leading to increased levels of c-Jun and c-Jun phosphorylation (53, 54).

## BCL10 filament formation

CARD11 fragments that include the CARD, LATCH and portions of the Coiled-coil domain have been shown *in vitro* to nucleate the formation of helical filaments of Bcl10 (55, 56). These Bcl10 filaments assemble through interactions between Bcl10 CARD domains and polymerize in a unidirectional manner (56). MALT1 and TRAF6 cooperatively associate with the Bcl10 filament (56) and stimulate MALT1 proteolytic activity (55). Bcl10 mutations that affect filament formation perturb NF-κB activation by overexpressed Bcl10 in cells (55), implicating these structures in CARD11 signaling. However, further work is needed to bolster the importance of Bcl10 filaments in antigen receptor signaling through CARD11 *in vivo* at physiological levels of expression, resolve how Bcl10 and MALT1 ubiquitinylation is accommodated or promoted by Bcl10 filament formation, and explain the mechanistic relationship between Bcl10 filament formation and other steps in CARD11 signaling that require the L3, SH3, L4, and GUK domains, which are required for physiological CARD11 signaling but are not required to nucleate Bcl10 filaments.

## Modulation and termination of CARD11 signaling

Multiple mechanisms have evolved to tune and terminate CARD11 signaling to ensure appropriate pathway output and avoid pathological immune cell activation and proliferation. First, cofactor association to CARD11 is regulated. The E3 ligase RNF181 limits the steady-state level of Bcl10 through K48-linked ubiquitinylation and degradation, thereby limiting the amount of Bcl10 that can bind CARD11 (57). Once signaling initiates, the kinesin GAKIN competes with Bcl10 for binding to CARD11 and limits the dwell time of CARD11 at the immune synapse (58). The phosphatase PP2A removes an activating phosphate from CARD11, which limits cofactor binding to CARD11 (59). Once CK1α is recruited to CARD11 in a step required for signaling, it phosphorylates CARD11 to inhibit the extent of CARD11 signaling (23).

Second, cofactors that associate with CARD11 in a signal-inducible manner rapidly dissociate from CARD11 during the initial ~60 minutes after receptor triggering. The mechanisms of complex disassembly remain poorly defined, but disassembly limits the generation of CARD11-promoted signaling intermediates. In some contexts, cofactors disassemble from CARD11 in an obligate step in productive signaling, such as in the assembly of complexes containing p62, Bcl10, MALT1, and the IKK complex that can prolong IKK activation (60, 61).

Third, key polyubiquitinylated signaling intermediates, including LinUb_n_-Bcl10, Ub_n_(K63)-Bcl10, and Ub_n_(K63)-MALT1 rapidly disappear as the result of the action of A20 (42), CYLD (46, 47), and likely other deubiquitinases. The removal of these potent intermediates attenuates the extent and duration of IKK complex activation. Fourth, CARD11 and Bcl10 are themselves degraded to limit signaling, and perhaps make cells refractory to immediate re-initiation of the CARD11-dependent pathway (15, 62–66).

## Somatic gain-of-function CARD11 mutations in lymphoma

**Figure 3:**
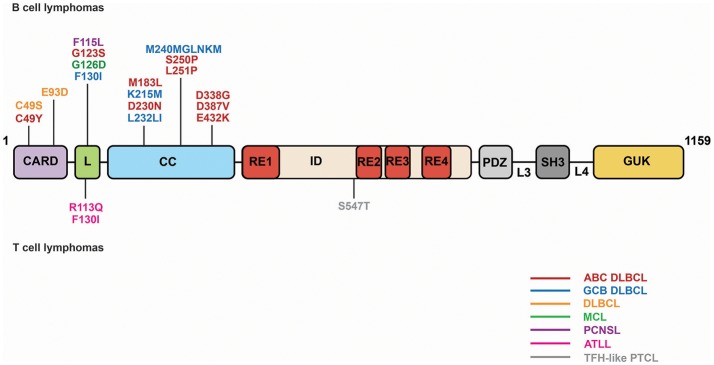
Lymphoma-associated CARD11 mutations. Mutations are depicted that have been validated to have at least a 3-fold increase in CARD11 signaling in a quantitative signaling assay. ABC, Activated B Cell-like; DLBCL, Diffuse Large B Cell Lymphoma; GCB, Germinal Center B cell; MCL, Mantle Cell Lymphoma; PCNSL, Primary Central Nervous System Lymphoma; ATLL, Acute T cell Leukemia/Lymphoma; TFH, Follicular T helper; PTCL, Peripheral T cell Lymphoma.

The dysregulated, constitutive signaling to NF-κB observed in several types of leukemia and lymphoma endows the transformed cells with a proliferative and survival advantage through the induction of pro-proliferative and anti-apoptotic NF-κB gene targets (40 67–69). Leukemias and lymphomas exploit a variety of strategies of genomic alteration to achieve constitutive NF-κB activity. The regulation of CARD11 activity by an internal autoinhibitory domain makes CARD11 highly susceptible to mutations that can cause GOF signaling independent of upstream antigen receptor engagement. GOF CARD11 mutations occur in approximately 10% of cases of the Activated B Cell-Like (ABC) subtype of Diffuse Large B Cell Lymphoma (DLBCL) (70), but they have also been observed in other DLBCL subtypes (71–76), as well as in Acute T-cell Leukemia/Lymphoma (77), Sézary syndrome (78, 79) Mantle Cell Lymphoma (80), and Angioimmunoblastic T-cell lymphoma (81). In many cases, however, the signaling potency of CARD11 alleles found in patient biopsies has not been thoroughly characterized, or confirmed to be required for aberrant proliferation, as has been done for many CARD11 mutations found in ABC DLBCL. Figure 3 depicts lymphoma-associated mutations that have been directly shown to potently increase CARD11 signaling.

**Figure 4:**
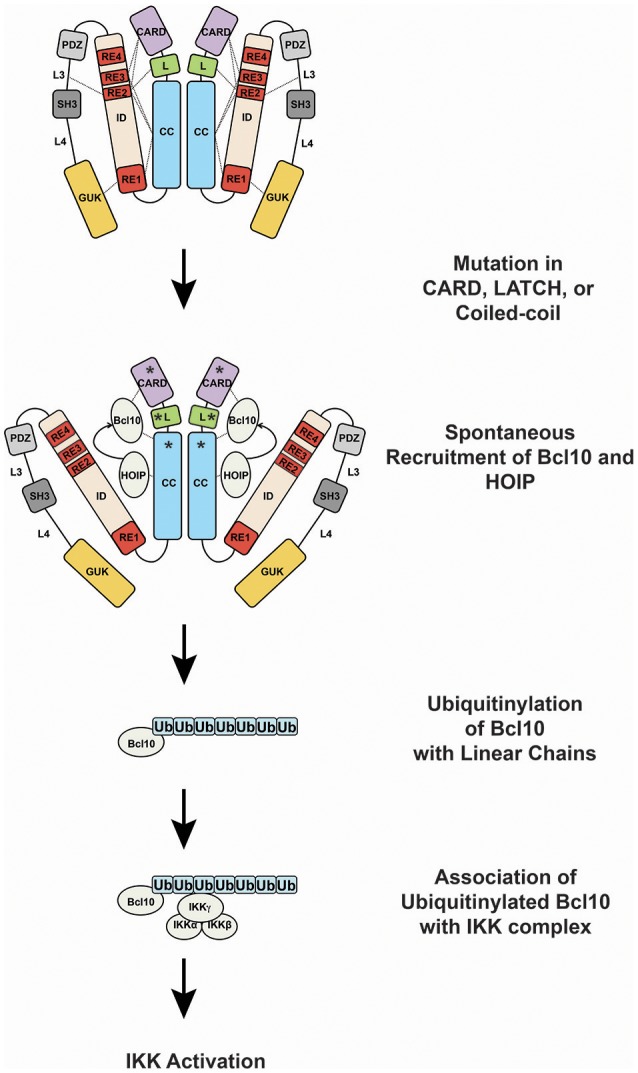
Oncogenic CARD11 signaling. Lymphoma-associated gain-of-function mutations in the CARD, LATCH, and Coiled-coil are represented by asterisks.

GOF CARD11 mutations cause constitutive hyperactive CARD11 signaling by bypassing the action of the four REs in the ID that normally keep CARD11 basally inactive (13, 22, 31, 82) (Figure 4). Mutations in the CARD, LATCH, and Coiled-coil of CARD11 disrupt the function of multiple REs to allow partial conversion of CARD11 to an open, active state that can recruit Bcl10 (31, 82) and HOIP (22), but not other factors recruited during normal antigen receptor signaling, including TAK1, TRAF6, IKKγ, and Caspase-8 (31, 82), at least for the mutants that have been characterized. The spontaneous recruitment of Bcl10 and HOIP to GOF CARD11 variants leads to the spontaneous generation of LinUb_n_-Bcl10, the levels of which appear to determine the quantitative output of NF-κB activation (22). Potent GOF mutations in the CARD, LATCH, and Coiled-coil can enhance basal CARD11 signaling by 80- to 160-fold (31, 82), and they appear to do so in part by interfering with inhibitory intramolecular interactions mediated by multiple REs (13). Potent GOF point mutations do not occur in the ID itself, due to the redundant action of the four REs; three or more REs would have to be disabled to achieve a comparable level of dysregulated signaling (12). For ABC DLBCL cells, the quantitative degree to which a GOF CARD11 allele activates NF-κB largely correlates with its ability to support aberrant cell proliferation (31, 70). However, the dysregulation of B cell proliferation *in vivo* by a CARD11 GOF allele requires both NF-κB and JNK activation (83). CARD11 GOF mutations in lymphoma occur in the presence of many other genomic alterations. While overexpression of an extremely potent CARD11 GOF allele is sufficient to cause lethal B cell proliferation (83), in human lymphomas it is likely that multiple genomic lesions cooperate with a GOF CARD11 mutation to maintain the proliferation and survival of the transformed cells.

## Germline CARD11 mutations in primary immunodeficiency

One of the most exciting recent developments in the study of CARD11 has been the recognition of germline CARD11 mutations in primary immunodeficiency. Three different forms of primary immunodeficiency (PID) have been described so far that result from germline mutations in the CARD11 gene, (1) CARD11 deficiency, (2) BENTA disease, and (3) Immunodeficiency with atopy.

**Figure 5:**
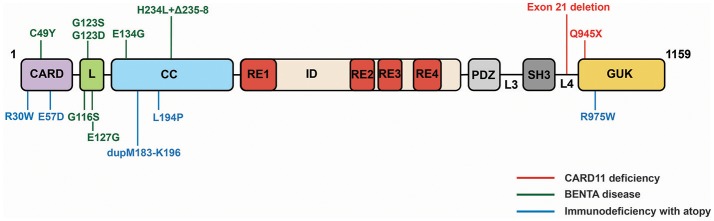
CARD11 mutations identified in three classes of primary immunodeficiency.

CARD11 deficiency caused by homozygous LOF mutations in CARD11 was first reported in two studies in 2013 (84, 85). These patients, who presented with severe *Pneumocystis jirovecii* infections as infants, displayed normal T and B cell counts but hypogammaglobulinemia, deficits in mature or differentiated B and T cells (CD4 and CD8), reduced Treg numbers, and defective B and T cell activation *in vitro*. The homozygous mutations found in these patients include a deletion of exon 21 that results in a lack of detectable CARD11 expression (85) and a premature stop codon at glutamine 945 in the GUK domain (84) (Figure 5). Notably, family members that are heterozygous for these alleles do not present with immunodeficiency.

BENTA disease (B cell Expansion with NF-κB and T cell Anergy), caused by heterozygous GOF CARD11 mutations, has been described in 16 patients so far beginning in 2012 (86–90). BENTA patients experience recurrent ear, sinopulmonary, and viral infections (molluscum contagiosum, BK virus, Epstein-Barr virus), and exhibit a profound expansion in the number of B cells, a skewing of B cells toward transitional states, an unresponsiveness of T cells to antigen, and a poor antibody response to pneumococcal and meningococcal capsular polysaccharides. BENTA-associated mutations are located in the CARD (C49Y), LATCH (G116S, G123S, G123D, E127H), and Coiled-coil (E134G, H234L+Δ235-8) domains (Figure 5). Some (C49Y, G123S, G123D) are identical to those found in DLBCL. The alleles induce constitutive NF-κB activation in lymphocytes, presumably through the same signaling intermediates discussed above, but it remains unclear precisely how their constitutive signaling results in disparate effects in different immune cell subtypes.

Immunodeficiency with atopy, caused by heterozygous, LOF CARD11 mutations that appear to act as strong dominant negative alleles, was reported in 2017 by two studies (91, 92). In the five families described so far, affected individuals experience severe atopic dermatitis, recurrent pneumonia, and other upper respiratory tract infections, asthma, and food allergies with varying severities. While patient B cells exhibit mild defects in antigen-induced activation, patient T cells display reduced activation and proliferation *in vitro*, consistent with a poor T cell response to prior antigen exposure. Patients also display elevated serum IgE levels but low-to-normal levels of other Ig classes. CARD11 mutations that cause Immunodeficiency with atopy have been found in the CARD (R30W, E57D), Coiled-coil (L194P, dupM183-K196), and GUK (R975W) (Figure 5). E57D and L194P have been shown to interfere with recruitment of Bcl10 and MALT1 to CARD11 following TCR signaling (91), while R30W appears to have a milder effect on recruitment of these cofactors to CARD11 (92). This class of dominant negative mutants has also been shown to disrupt CARD11 signaling to mTOR and to the activation of MALT1 protease activity (91).

## Current key questions and opportunities

### What is the 3D structure of CARD11?

A thorough understanding of how CARD11 is kept inactive prior to signaling, and how CARD11 converts to an open, active scaffold will require determination of the three-dimensional structure of CARD11 in “closed” and “open” states. Structural studies so far have solved the structure of the CARD domain of CARD11 and have modeled how the CARD11 CARD can nucleate the formation of Bcl10 filaments (55, 56, 93, 94). However, the structure of 90% of the protein is unknown. Structural information will be invaluable for understanding how the multiple domains in CARD11 signal to its targets and how GOF and LOF mutations induce or disrupt CARD11 activity.

### How does CARD11 mediate IKK complex activation?

It remains unclear precisely how CARD11 signaling results in the activation of IKK kinase activity. The generation of ubiquitinylated Bcl10, MALT1, and IKKγ species is thought to induce a network of interacting proteins, in which ubiquitin chains are recognized by specific domains within signaling cofactors. This web of intermolecular binding can induce the proximity and clustering of IKK complexes, but how kinase activity is induced under physiological conditions, and whether other requisite components have yet to be discovered are unclear. It should also be explored whether CARD11 simply recruits enzymes (E3 ligases, kinases, protease) to their substrates or whether CARD11 binding plays a more active role in allosteric regulation of catalytic activity or substrate competency.

### What is the physiological function of the four REs?

Although it is clear that the four REs within the ID function cooperatively to keep CARD11 inactive prior to signaling, it remains unclear why the protein has evolved this unique array of redundant repressive elements. RE redundancy does prevent unwanted GOF mutations from occurring in the ID, but it does not prevent their occurrence in the CARD, LATCH, and Coiled-coil. It is possible that the REs determine the kinetics of CARD11 “activation” during signaling, or the kinetics by which CARD11 returns to the basal inactive state following signaling, but further studies are needed to test these hypotheses.

### How precisely do CARD11 GOF and LOF alleles cause immunodeficiencies of variable phenotype?

The discovery of patients with germline CARD11 LOF and GOF mutations provides exciting opportunities for obtaining insight into the molecular mechanisms of CARD11 signaling and the cellular interplay of immune cell subtypes affected by CARD11 dysfunction. It remains unclear why some CARD11 LOF mutations are dominant negative and manifest disease when heterozygous, while other CARD11 LOF mutations manifest disease only when homozygous. In addition, it is not firmly established whether all disease-associated CARD11 alleles affect signaling to mTOR and JNK, or which dysregulated pathways downstream of CARD11 are responsible for which disease manifestations. Also unknown is whether modifier genes in the patients studied have influenced their presentation, since only a small number of patients have been identified so far. It will be interesting to see whether additional CARD11 alleles will be discovered in the human population, leading to variable phenotypes of immunodeficiency and atopy.

### What other signaling pathways depend on CARD11?

Several studies have implicated a role for CARD11 in pathways distinct from antigen receptors, including those emanating from activating NK cell receptors (95–97), OX40 (98), and the IL-2 receptor (99). The mechanistic role of CARD11 in these pathways deserves further study. It is possible that additional signaling pathways will be identified that rely on CARD11 activity, and if dysregulated, may contribute to human disease.

## Author contributions

All authors listed have made substantial, direct, and intellectual contribution to the work and approved it for publication.

### Conflict of interest statement

The authors declare that the research was conducted in the absence of any commercial or financial relationships that could be construed as a potential conflict of interest.
